# Beneficial Effects of Natural Bioactive Compounds on Eye Health: A Narrative Review

**DOI:** 10.3390/ijms27104592

**Published:** 2026-05-20

**Authors:** Sandun De Silva, Baojun Xu

**Affiliations:** 1 Food Science and Technology Program, Department of Life Sciences, Beijing Normal-Hong Kong Baptist University, Zhuhai 519087, China; desilva15at@ags.ruh.ac.lk; 2 Department of Food Science and Technology, Faculty of Agriculture, University of Ruhuna, Mapalana, Kamburupitiya 81100, Sri Lanka

**Keywords:** eye health, bioactive compounds, carotenoids, polyphenols and flavonoids, omega-3 fatty acids, oxidative stress and inflammation, age-related macular degeneration

## Abstract

Ocular diseases like age-related macular degeneration (AMD), diabetic retinopathy (DR), glaucoma and cataracts are major causes of visual impairment all over the world and are closely linked to oxidative stress, inflammation and mitochondrial dysfunction. This narrative review critically summarizes the available evidence on how various natural bioactive compounds, such as carotenoids, polyphenols, flavonoids, omega-3 fatty acids and botanical extracts, can affect important molecular pathways associated with ocular degeneration. Their antioxidant, anti-inflammatory, anti-angiogenic and neuroprotective properties are given particular emphasis, especially regarding the Nrf2, NF-κB and VEGF signaling pathways. This review is different from past reviews that simply discuss the potential of bioactives in the general nutritional context; rather, it unfolds the disease-specific mechanisms and compound-specific molecular actions and gives special attention to recent advances in nano-delivery systems and precision nutrition strategies to increase the bioavailability and therapeutic targeting of these nutrients in the eyes. Moreover, it offers a framework for a comparison of evidence between preclinical and clinical studies, as well as identifying current translational gaps, including limited bioavailability and a lack of long-term clinical trials, and suggesting future directions such as genotype-guided nutrition and microbiome-informed interventions. In general, this review provides a mechanistic and translational overview of how dietary bioactive compounds relate to eye health and offers the perspective of their possible use in prevention and complementary treatment for vision-related diseases.

## 1. Introduction

Visual impairment is a major global health problem with an increasing prevalence due to population ageing, higher incidence of metabolic diseases such as diabetes, and prolonged exposure to digital devices. Major vision-threatening conditions such as glaucoma, cataracts, age-related macular degeneration (AMD), diabetic retinopathy (DR), uveitis, retinal detachment, etc., contribute significantly to functional disability and reduced quality of life [1,2]. The expected increase in these disorders highlights the urgent need for effective, safe and long-term preventive and therapeutic strategies for different populations. Oxidative stress, chronic inflammation, mitochondrial dysfunction, dysregulated angiogenesis and metabolic imbalance are highly associated with the molecular-level pathogenesis of these ocular diseases. Nuclear factor erythroid 2–related factor 2 (Nrf2), nuclear factor kappa B (NF-κB), vascular endothelial growth factor (VEGF), and apoptosis-related cascades are the major signaling pathways that play central roles in mediating retinal degeneration, vascular damage, and neuronal loss. Interconnected mechanisms are important targets for intervention. Natural bioactive compounds have emerged as promising candidates to modulate such molecular drivers. Bioactive compounds are non-nutritive components of plant, microbial and algal sources that have biological effects other than basic nutrition [2]. The major classes are polyphenols, flavonoids, carotenoids, terpenoids, alkaloids, phytosterols, omega-3 polyunsaturated fatty acids (PUFAs), vitamins and minerals [3]. These compounds possess varied pharmacological properties, like antioxidant, anti-inflammatory, anti-angiogenic and neuroprotective activities. The effect of bioactive compounds is not generic health promotion but, importantly, the specific modulation of molecular pathways. For instance, polyphenols induce Nrf2-mediated antioxidant pathways and inhibit inflammatory responses via NF-κB. Carotenoids (e.g., lutein and zeaxanthin) protect retinal cells by filtering blue light and decreasing oxidative damage. Omega-3 PUFAs modulate inflammatory mediators and vascular function. Furthermore, bioavailability and therapeutic potential are increased by synergistic interactions among bioactives in whole extracts and improvements in delivery systems (e.g., nano-formulations). Natural bioactives are a promising approach for the prevention and management of chronic ocular diseases because of their structural diversity, multi-target activity and relatively low toxicity compared to synthetic drugs [4,5,6]. This review (2008–2026) aims to comprehensively evaluate the beneficial effects of natural bioactive compounds on eye health, focusing on (i) major classes of bioactives relevant to ocular function, (ii) their molecular mechanisms of action, (iii) evidence from preclinical and clinical studies, and (iv) current limitations and future research directions.

## 2. Methodology

**Figure 1:**
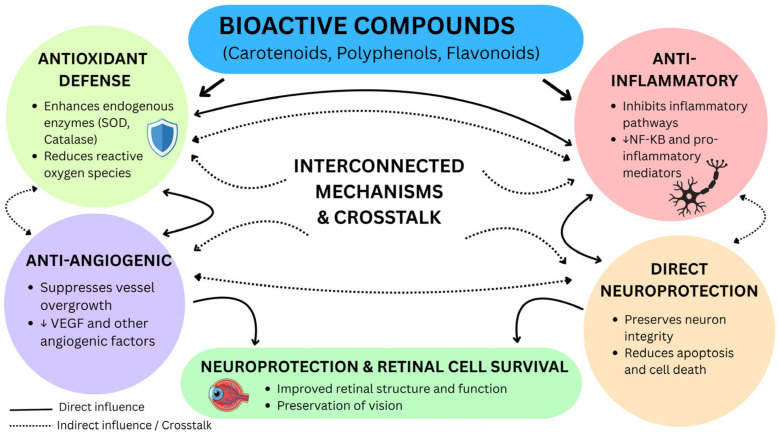
Interconnected mechanisms of bioactive compounds in ocular health: antioxidant, anti-inflammatory, anti-angiogenic, and neuroprotective crosstalk.

A comprehensive literature search was conducted using multiple scientific databases, including PubMed, Scopus, Web of Science, and Google Scholar, to identify relevant studies on the beneficial effect of natural bioactive compounds on eye health. We selected articles for use by combining keywords for the selection strategy. The keywords included “bioactive compound and eye health,” “lutein,” “zeaxanthin,” “polyphenols,” “omega-3 fatty acids,” “curcumin,” “diabetic retinopathy,” “age-related macular degeneration,” “dry eye,” and “ocular oxidative stress.“ The inclusion criteria for selecting articles were as follows: (1) studies published from 2008 to 2026, (2) articles available in English, and (3) studies focusing on bioactive compounds on eye health. The method used ensured the inclusion of high-quality research in this review, with its focus on the beneficial effects of natural bioactive compounds on eye health. The overall mechanism of action of bioactive compounds in retinal protection is depicted in Figure 1, which demonstrates the intricate mechanisms of antioxidant, anti-inflammatory, anti-angiogenic and neuroprotective effects in ocular health and retinal degeneration prevention.

## 3. Pathophysiology of Major Eye Diseases

### 3.1. Oxidative Stress and Inflammation in Eye Pathology

Oxidative stress is the central pathogenic mechanism in major eye diseases. The eye lens constantly faces oxidative stress from inflammation as a result of endogenous and ionizing X-rays and gamma rays as well as exogenous sources. It mainly occurs when reactive oxygen species (ROS) like hydrogen peroxide and hydroxyl radicals overcome the eye’s antioxidant defenses, leading to cellular damage. Reperfusion of the eye can cause ocular pathologies such as age-related macular degeneration (AMD), diabetic retinopathy/glaucoma, cataracts, and dry eye. Oxidative stress in ocular tissues is triggered by a few factors, such as exposure to light, high oxygen demand, old age, diabetes and chronic inflammation. The photooxidation of chromophores by ultraviolet (UV) and blue light exposure produces reactive oxygen species (ROS) in the lens and retina. Moreover, the retina is extremely metabolically active and demanding, with a high metabolic rate and oxygen consumption, which leads to a higher production of mitochondrial ROS during cellular respiration. The tissues of the eyes have highly regulated levels of oxygen and antioxidants to prevent damage from oxidative stress; however, over time these protective mechanisms are weakened in the healthy eye and are further compromised in pathological conditions, including diabetes and inflammation [7,8,9]. ROS production oversteps antioxidant capacity, and oxidative damage to lipids, proteins, and DNA occurs, directing cellular dysfunction, apoptosis, and chronic inflammation [10]. Eye inflammation/ocular inflammation results in redness, swelling, and pain due to irritation, as well as core autoimmune conditions. Inflammation and oxidative stress are interconnected with one another, because ROS inflammation is single and amplifies ROS production, driving diseases like AMD, DR, glaucoma, and dry eye [1,11]. Chronic inflammation also produces pro-inflammatory cytokine production (IL-6, IL-8, TNF-α) and activation of nuclear factor kappa B (NF-κB) signaling [12]. In the retina and retinal pigment epithelium, mitochondrial reactive oxygen species and lipofuscin gathering activate NLRP3-mediated NF-κB and VEGF signaling, leading to neovascularization and retinal pigment epithelial cell death, AMD and DR [13]. In the lens, ultraviolet-induced hydrogen peroxide promotes inflammatory cytokines such as IL-1β and TNF-α, resulting in crystallin aggregation in cataracts [14]. The abnormally high oxygen use of the retina (~65% cerebral equivalent) produces mitochondrial superoxide, which is enhanced by the blue light photooxidation of lipofuscin/A2E in RPE, leading to the NLRP3 inflammasome activation → IL-1β/IL-18 → NF- KB/VEGF cascade in AMD. DR hyperglycemia stimulates PKC/AGE-RAGE → NADPH oxidase → endothelial BRB rupture, and UV-H_2_O_2_ in cataracts oxidizes crystalline to insoluble aggregates. These are cross-linked ROS–inflammation cycles, which have been confirmed in ARPE-19 and Abca4-/- models as well as in human vitreous models and constitute conserved therapeutic targets [12,15,16].

### 3.2. Common Molecular Pathways Targeted by Bioactive Compounds

The protective abilities of bioactive compounds in the eye are mediated through multiple interconnected molecular pathways, such as antioxidant defense mechanisms, anti-inflammatory signaling pathways, regulation of angiogenesis, and neuroprotective mechanisms. These pathways are key targets of natural bioactive compounds like polyphenols (anthocyanins), carotenoids (lutein), and omega 3 fatty acid in preventing oxidative stress and inflammation of the eye [1,17]. The ocular protective properties of bioactive compounds are mediated by several molecular mechanisms, such as antioxidant effects through direct scavenging of reactive oxygen species (ROS) and activation of the Nrf2 signaling pathway, anti-inflammatory effects through modulation of the NF-κB and MAPK pathways, anti-angiogenic effects through the regulation of vascular endothelial growth factor (VEGF), and neuroprotective effects through anti-apoptotic signaling and the up-regulation of neurotrophic factors [6,13,18]. When these phytochemicals activate Nrf2, it moves to the nucleus and, in turn, induces antioxidant genes and inhibits the canonical pathway of NF-κB indirectly. The IKK complex (mainly IKKβ) phosphorylates IκB, which is then degraded, and NF-κB enters the nucleus to express pro-inflammatory genes; Nrf2 is an anti-inflammatory protein that interferes with this process, either by competing with upstream regulators such as Keap1 or by directly inhibiting IKKβ phosphorylation. This crosstalk decreases the levels of ROS, stabilizes IkB and inhibits the translocation of NF-kB, which has been demonstrated in neuroinflammation and cancer research [19,20,21,22]. Recent high-impact publications showcase how bioactive components such as polyphenols and carotenoids interact with molecular targets, such as Nrf2 activation and NF-κB repression (via IKKβ inhibition), as well as addressing delivery bottlenecks through combinations. For example, nano-delivery, such as through lipid nanoparticles and liposomes, increases the bioavailability of curcumin/resveratrol 2–3× fold, preserving synergies with Nrf2 to protect against oxidative stress and inflammatory eye diseases. Co-delivery nano emulsions for hydrophilic/hydrophobic pairs also boosts antioxidant effects, overcoming GIT instability and low solubility, and is suitable for vegan nutraceuticals from indigenous pulses. These innovations reveal ongoing standardization challenges but set combinational systems to revolutionize clinical translation [23,24,25]. Nano-delivery transforms translation: factorial-optimized curcumin/resveratrol lipid nano capsules (2026) offer 11× plasma Cmax and sustained RPE delivery, with Nrf2/HO-1 activation, and co-encapsulating lutein overcomes parallel solubility obstacles. These plant systems (*Mesona chinensis* polysaccharides) enhance GIT stability threefold, providing therapeutic Nrf2-IKKβ regulation that cannot be achieved with free compounds and serving as a suitable option for vegan formulations with pulses [26,27,28,29].

## 4. Major Classes of Bioactive Compounds Relevant to Eye Health

### 4.1. Carotenoids

Carotenoids are yellow, red, and orange pigments synthesized by plants and algae. Animals and humans cannot synthesize carotenoids; these compounds are obtained from diet. These compounds help to prevent cardiovascular diseases, certain types of cancer, eye-related diseases, and light-induced skin damage. In the eye, the retina contains the macula, which is supported by the carotenoids lutein, zeaxanthin, and meso-zeaxanthin [26]. The daily intake of 10 mg of lutein and 2 mg of zeaxanthin is recommended for ocular health in humans [14]. Green leafy vegetables as well as orange and yellow fruits and vegetables commonly include carotenoids. Carrots, kale, orange peppers and spinach include these compounds. Lutein and zeaxanthin have a higher antioxidant effect. Lutein helps to protect the retina against the phototoxic activity of blue light [8] because it reduces phototoxic damage to photoreceptor cells. Lutein can defeat the pro-inflammatory cytokine cascade and the motion of the dictation factor nuclear factor-kB (NF-kB). Studies have also focused on lutein’s effectiveness in lowering the generation of reactive oxygen species (ROS), decreasing levels of inducible nitric oxide synthase (iNOS), and influencing the stimulation of the complement system [3]. Carotenoids, including lutein, help protect against AMD by minimizing oxidative stress in the retina through several pathways. Their filtering capability lessens the amount of high-energy light reaching the photoreceptors. In adding, carotenoids function as straight antioxidants and can initiate cellular pathways that lead to indirect antioxidant benefits. More research is needed to fully realize the specific cellular signaling pathways activated by lutein in the eye [26]. Commonly known as the “eye vitamin,” lutein is vital for shielding the eyes from conditions such as age-related macular degeneration (AMD), diabetic retinopathy, and cataracts. Its antioxidative, anti-inflammatory, and blue-light-filtering qualities protect both the lens and retina, emphasizing the importance of a sufficient diet. Other carotenoids show promise: *β*-cryptoxanthin shows antioxidant properties and converts to retinol, and crocin from saffron demonstrates neuroprotective effects in preclinical findings and has enriched visual function parameters in small AMD trials [30].

The build-up of lutein, zeaxanthin and meso-zeaxanthin is unique in the macula (1000× plasma levels) and occurs through special transport pathways that differ from other phytochemicals. Circulating via HDL/LDL, xanthophylls cross RPE via SCARB1/CD36 receptors, bind IRBP in interphotoreceptor matrix, and enter photoreceptors/Henle fibers through GRAMD1B lipid transfer, preventing peripheral enzymatic cleavage: dietary micelles → intestinal SR-B1 → chylomicrons → liver → HDL → RPE STRA6/RBP-independent apical uptake → RPE65 enzymatic lutein-to-meso-Z conversion. Foveal gradient (Z > L > MZ peripherally → MZ-center dominance) replicates sequential isomerization + neuronal sequestration, presenting a photooxidative defense absent in BRB-limited polyphenols [31,32,33,34]. Zeaxanthin and meso-zeaxanthin are essential to visual health even though they are not found in large amounts in the diet as compared to lutein. In mid-macular areas, the predominant form of zeaxanthin, which filters blue light (460 nm peak) and quenches singlet oxygen in lipid rafts, is predominant, with the oxidative risk-prone lipid epicenter (the highest cone density) being foveal. The supplementation (10 mg MZ + 2 mg Z) substantially increases the MPOD over 18 months and enhances glare recovery and mesopic contrast sensitivity in glaucoma patients—effects remaining despite the insignificant contribution of grapes (under 0.1 mg/100g carotenoids). Their non-interchangeability with lutein can be explained by their unique foveal sequestration through GRAMD1B/IRBP [35,36,37,38]. 

Though, in human diets, carotenoids are predominantly plant-derived (or subject to synthesis), a small fraction of other phenomena is considered to contain them, such as aphids, ladybugs, etc. Microbial carotenoid intermediates play indirect roles in food webs (ingestion is mostly microbial), and insect-derived preparations have been used to biomimetically nanoencapsulate various biomolecules. This biosynthesis diversity can support sustainable production of vegan foods but does not alter the primary pathways of RPE transport through HDL/SCARB1 pathways reported to support transport of xanthophylls in plants [39,40,41].

### 4.2. Omega-3 Fatty Acids

Omega-3 fatty acids cannot be produced in the body; they are essential fatty acids. Long-chain omega-3 fatty acids docosahexaenoic acid (DHA) and eicosatetraenoic acid (EPA) are derived from oily fish and marine food [42]. Omega-3 fatty acids affect ocular health, including functions in retinal arrangement, inflammatory parameters, and intraocular pressure modulation [43]. Omega-derived epoxy eicosanoids exhibit cardioprotective, vasodilatory, anti-inflammatory, and anti-allergic activities [43]. Neuroprotection D1 (NPD1), derived from DHA, protects RPE and photoreceptors from oxidative apoptosis, inhibits pro-inflammatory signaling, and promotes cell survival. Clinical associations link enhanced omega-3 intake with reduced AMD risk and enriched dry eye outcomes [44,45]. EPA/DHA generate specialized pro-resolving mediators (SPMs) like resolvins (RvD1-6), protectins (neuroprotectin-D1), and maresins, which actively resolve inflammation by clearing neutrophils/apoptotic cells, inhibiting NF-κB/TLR4, reducing IL-6/IL-8/TNF-α from microglia, and suppressing arachidonic acid-derived pro-inflammatory prostaglandins/leukotrienes [22,23]. Specialized pro-resolving mediators (SPMs) derived from omega-3s such as resolvins (RvD1, RvE1) and neuroprotectins (NPD1) actively facilitate the resolution of inflammation in a temporal biochemical cascade, and not simply by passive reduction [46]. Phase 1 (Initiation, 0–6h): DHA is oxidized by enzymes, 15-LOX/5-LOX, into RvD1, which binds to DRV1/GPCR32 receptors on microglia/macrophages, causing NF-Kb/TNF-a signaling to be inhibited and triggering efferocytosis [47]. Phase 2 (Peak Resolution, 6–24h): NPD1 (via DHA through 15-LOX) peaks, causing retinal pigment epithelium (RPE) RXFP1/ALX/FPR2 receptors to up-regulate Bcl-2/Bcl-xL, inhibiting Bax/caspase-3 apoptosis and promoting IL-10. Phase 3 (Tissue Repair, >24 h): RvD1/RvE1 promotes neutrophil apoptosis and monocyte phagocytosis, which repairs corneal/RPE barrier integrity through VEGF-A regulation. This organized sequence validates DR/AMD model SPMs as active termination signals, which interact with Nrf2 flavonoids to provide overall ocular protection [23,24,48].

### 4.3. Polyphenols

Polyphenols are divided into two parts: flavonoids and nonflavonoids. Nonflavonoid polyphenols include phenolic acids (ferulic acid), stilbenes (resveratrol), curcuminoids (curcumin), and lignans. Flavonoids are further subdivided into six principal subgroups: flavones, flavonols, flavanols, flavanones, isoflavones, and anthocyanins [1]. Coffee, tea, cocoa, and apples are the main food sources that provide polyphenols. Polyphenols protect against retinal degenerative diseases like retinitis pigmentosa, where mutations in retinal proteins cause photoreceptor cell death and vision loss [49].

#### 4.3.1. Flavonoids

Flavonoids show antioxidant properties in vitro and act as redox modulators *in vivo*, supporting retinal and neuronal tissues and reducing oxidative damage to the eye’s lens. Evidence suggests they stabilize collagen and improve microvascular integrity, potentially slowing retinopathies and glaucoma. Despite promising findings regarding their anti-angiogenic and anti-inflammatory effects on age-related macular degeneration (AMD), well-designed randomized controlled trials are needed. Diets rich in flavonoid-packed fruits and vegetables have been associated with reduced risk of various vision disorders, including diabetic retinopathy, glaucoma, and cataracts [17,18]. Quercetin has also produced a protective effect against oxidative stress and its consequences on photoreceptor cells, resulting from the reaction of ATR with phosphatidylethanolamine, producing bis-retinoid photoreactive species. Mechanisms involved in the antioxidant activity of polyphenols include the suppression of ROS formation, thus reducing oxidative damage. The mechanism by which ROS formation is reduced involves phosphorylation of Nrf2 residues, resulting in nuclear accumulation [49,50,51]. 

Newer flavonoids such as pinocembrin, naringenin and eriodictyol provide compound-specific mechanistic evidence that can be used to take the discussion past generalized polyphenols, directly to the Nrf2/HO-1 axis, apoptosis control, and mitochondrial protection of oxidative ocular models. Pinocembrin (25 μM) protects SH-SY5Y neuronal cells against the effects of H_2_O_2_-induced mitochondrial depolarization, lipid peroxidation and TCA cycle disruption (aconitase/α-KGDH inhibition) via Erk1/2-mediated Nrf2 nuclear translocation—effects totally blocked by Nrf2 siRNA. Naringenin also rescues mitochondrial complexes I/V, GSH homeostasis, and caspase-3 apoptosis in RPE/stressed neurons by Nrf2-mediated Erk phosphorylation and prevents retinal endothelial/RGC cells from high-glucose inflammation by HO-1 up-regulation. Such dissectioned processes place flavanones as selective Nrf2 agonists to enhance AMD-RPE survival and glaucoma-RGC neuroprotection, as opposed to general antioxidants [52,53,54,55].

#### 4.3.2. Anthocyanin

Flavonoid pigments show polyphenolic abundance in berries [46]. Anthocyanins generate red/purple pigments and stimulate eye wellness through antioxidative, anti-inflammatory, and vasoprotective effects. They include the scavenging of reactive oxygen species (ROS) to protect the retinal cells, the regeneration of rhodopsin to increase dark adaptation, ciliary relaxation to relieve myopia and fatigue, and retinal microcirculation through activating eNOS [24,25]. Anthocyanins counteract the effects of ROS by direct scavenging and Nrf2 pathway activation, which increases HO-1, SOD, and GSH-Px to reduce lipid peroxidation and DNA damage in photoreceptors and RPE. This prevents blue light/H_2_O_2_ oxidative damage in AMD/DR models, maintains cell survival and MPOD, and acts synergistically with lutein to offer total retinal protection [56]. The synergies (polyphenols + carotenoids) are mechanistically explained by the convergence of pathways and enhanced bioavailability and not necessarily by sequential signaling alone. As an example, Nrf2 activation by carotenoids (e.g., lutein/zeaxanthin) converges with the NF-KB/IKKβ inhibition of polyphenols (e.g., curcumin/resveratrol) at common ROS-sensitive nodes, resulting in multiplicative antioxidant effects. Co-encapsulation in nano emulsions resulting in sequential modulation takes place secondarily, e.g., primary Nrf2-initiated ROS quenching allows prolonged NF-KB blockade, whereas bioavailability synergy prevails in translation, with combinational lipid NPs overcoming GIT/BBB barriers to reach therapeutic levels of ocular concentrations otherwise accessible only singly [23,57]. This systematic interaction makes vegan multi-compound preparations superior in terms of AMD/DR intervention [24,46].

Precision extension of forward-looking precision: Novel combinational strategies combine these synergies with specific technologies. Cell-type-specific pathway regulation (RPE vs. RGC) using CRISPR-Nrf2 enhancers and flavonoid-NP conjugates, as well as microbiome-stratified urolithin-generating cohorts, optimize polyphenol conversion. AI-based PK modeling is used to optimize vegan nano emulsion ratios for each Nrf2-genotype/metabotype and changes multi-compound nutraceuticals into personalized precision therapies, with estimated 3–5× improvements in efficacy under monotherapy. Translational frontier Phase II trials of AREDS2 carotenoids + genotyped flavonoid-NPs are in progress [24,58].

#### 4.3.3. Resveratrol 

Another polyphenolic stilbene bioactive substance that is available in grapes and berries is resveratrol. Resveratrol assists in providing anti-oxidative, anti-apoptotic, anti-tumorigenic, anti-inflammatory, anti-angiogenic, and vasodilator effects to the eye [27,28]. Resveratrol also enhances retinal formations and increases blood flow, and thus it increases oxygen supply in parts of the eye that lack oxygen supply [59,60,61]. Resveratrol (*trans*-3,5,4′-trihydroxystilbene) activates SIRT1→p53/FoxO3a inhibition and Nrf2 via AMPK, penetrating the RGC layer for glaucoma neuroprotection. It is unique among polyphenols for BBB penetration (0.2 μM brain post-50 mg) and is prevalent in grape skins/red wine (0.1–5 mg/L) [61]. Tannic acid has beneficial effects on health, including the reduced production of UVB-induced interleukin 18 in human keratinocytes as well as anti-inflammatory properties [49]. 

Resveratrol stimulates SIRT1 to deacetylate p53/FoxO3a and up-regulate Ku70/Bcl-2, thereby inhibiting caspase-3/8/9-driven apoptosis and inhibiting TNF-a; IL-6/8/COX-2 via the activation of Nrf2/HO-1 inhibits ROS and lipid peroxidation in RPE during blue light or hyperglycemia, suppressing the down-regulation of HIF-1a/VEGF [62,63].

#### 4.3.4. Epigallocatechin Gallate (EGCG) 

Epigallocatechin gallate is the major catechin of polyphenol in green tea, black tea and apples [64]. It possesses protective effects through a variety of pathways, such as antioxidative, anti-inflammatory, neuroprotective, anti-angiogenic, and anti-apoptotic. EGCG can suppress lipid peroxidation and DNA damage in RPE during blue light/UVB stress, inhibit p38/ERK MAPK phosphorylation and VEGFA expression to inhibit CNV in wet AMD, and stabilize mitochondrial membrane potential by suppressing Bax translocation and caspase cascade activation in light-damage and RP models; systemic doses (25–50 mg/kg) can be used to inhibit hepatic oxidative load by increasing Nrf2/HO-1 signaling [64,65,66].

### 4.4. Ginsenosides

The ginsenosides family is a source of steroidal saponins, found in ginseng and American ginseng [67]. Ginseng offers protective effects against age-related macular degeneration (AMD), diabetic retinopathy, glaucoma and retinal damage due to light [68,69]. Ginsenoside Rg3 (S-Rg3) is a necessary bioactive agent for use in eye health. Ginsenosides have multiple pathways of retinal cytoprotection: compound K activates Nrf2 to induce HO-1/SOD/GSH-Px to counter H_2_O_2_-induced ROS/lipid peroxidation in RPE; Rb1 suppresses ARPE-19 VEGF release to inhibit CNV; Rg1 suppresses GCL/INL apoptosis by modulating Bcl-2/caspase-3 in db/db DR models; Re preserves photoreceptor loss by photooxidation [67,68,69,70,71,72].

### 4.5. Additional Bioactive Compounds from Botanicals

Curcumin, saffron, and *Ginkogo biloba*, like botanical compounds, help to prevent eye diseases. Turmeric (*Curcuma longa)* contains the bioactive compound curcumin. Curcumin has given protective effects on ocular health, due to its anti-inflammatory and antioxidant properties. Curcumin protected the retina in vivo, preserving retinal function, reducing oxidative stress, inflammation, and apoptosis, and slowing degeneration in AMD and light-induced retinal damage [1]. It controls inflammatory enzymes, including cyclooxygenase-2 (COX-2), lipoxygenase, and inducible nitric oxide synthase (iNOS), and suppresses the production of pro-inflammatory cytokines such as interleukins and monocyte chemoattractant protein (MCP). During inhibition of NF-κB signaling and regulation of molecular pathways involving AKT and NRF2, curcumin reduces oxidative stress and inflammatory genes in ocular tissues. Curcumin prevents age-related macular degeneration (AMD), diabetic retinopathy (DR), and cataract formation [73]. 

Saffron and its bioactive compounds, including crocin, crocetin, and safranal, possess potent retino-protective and neuroprotective properties. Crocin (1 mg/kg) reduced RGC apoptosis 45→28% in light-damaged rats; lutein (0.2 mg/kg diet) increased ONL thickness by 18% in Abca4-/- mice. Crocin activates the PI3K/AKT signaling pathway, thereby mitigating oxidative stress, inflammation, and apoptosis in retinal cells, protecting photoreceptors from light- and H_2_O_2_-induced damage. Saffron improves visual function, preserves retinal structure, and inhibits oxidative stress in mediated cell death through modulation of mitochondrial pathways, NF-κB signaling, and suppression of ROS and LDH [73,74].

*Ginkgo biloba* extract (GBE) has demonstrated promising potential in the management of glaucoma by enhancing ocular blood flow. Clinical assessments utilizing color Doppler imaging revealed that GBE increased the ophthalmic artery end diastolic velocity compared to placebos, suggesting improved ocular perfusion [67,75,76]. An overview of key bioactive compounds, including their sources, actions, and specific ocular conditions, are given in Table 1.

Saffron activates PI3K/Akt → Nrf2/HO-1 in photoreceptors, reducing H_2_O_2_-induced RGC apoptosis from 45% to 28% (0.1–1 μM, Annexin V/PI) via mitochondrial Bcl-2 stabilization. Curcumin inhibits IKKβ kinase activity (IC_50_~5 μM) and COX-2/iNOS in RPE, blocking NF-κB→IL-6/TNF-α while synergizing with lutein via Nrf2 convergence. *Ginkgo biloba* flavonoids enhance ophthalmic artery end-diastolic velocity by 23% (6.5→7.7 cm/s, *p* = 0.023 vs. placebo) through PDE inhibition/NO up-regulation, addressing glaucoma vascular deficits [78,79,80,81].

## 5. Mechanisms of Action

### 5.1. Antioxidant Effects

The antioxidant system mechanism involves controlling the production of ROS and the endogenous antioxidant enzymatic system, including catalase and glutathione peroxidase. The reinforcement of the endogenous antioxidant system of ocular tissues [82] can be achieved through nutrition and food supplementation, as well as through medical devices and antioxidant-based drugs. Exogenous plant derived antioxidants, water-soluble promoters of the endogenous antioxidant system, and lipophilic antioxidants are the three antioxidant molecules involved. Oxidative stress plays a central role in retinal degeneration due to the high oxygen consumption, rich polyunsaturated fatty acid content, and constant light exposure of the retina and retinal pigment epithelium (RPE) [83]. The major mechanisms of antioxidants are complete free radical scavenging, where the reactive oxygen species (ROS) of superoxide anions, hydroxyl radicals and hydrogen peroxide are neutralized by donating electrons or hydrogen atoms, thus preventing oxidative chain reactions [84,85]. By diminishing the level of ROS, antioxidants reduce lipid peroxidation, protein oxidation, DNA damage, and mitochondrial dysfunction in retinal and RPE cells. This protective measure preserves cell integrity, restrains apoptosis, and preserves vision, ultimately decelerating the progress of oxidative stress-induced retinal diseases. 

In addition to direct ROS scavenging, biological active compounds rehabilitate the Nrf2/Keap1 pathway. Carotenoids (lutein 10 mM) can induce nuclear translocation of Nrf2 in ARPE-19 cells and can up-regulate HO-1 (4.2×), NQO1 (3.8×), and GCLC via ARE-binding; polyphenols (quercetin 25 mM) can restore activities of the mitochondrial complexes I/II and GSH/GSSG ratios in RPE treated with H_2_O_2_. These transcriptional (not just stoichiometric) effects describe higher retinal protection with respect to synthetic antioxidants, with tissue-specific distribution showing lutein in RPE outer segments (520 nm peak), versus resveratrol and its photoreceptor preference [86,87].

The human eye has a complex defense mechanism to combat oxidative stress, integrating both enzymatic and non-enzymatic antioxidants to neutralize reactive oxygen species (ROS) and prevent cellular damage caused by metabolic processes, increased oxygen concentrations and exposure to light. The most important enzymatic antioxidants are superoxide dismutase (SOD), which was the first enzyme to be recognized as an antioxidant. The catalase (CAT) breaks down hydrogen peroxide to water and oxygen, especially protecting the lens against oxidative damage and acting as a backup in chronic exposure situations. Complementary to the glutathione system are glutathione peroxidases (GPXs) and peroxiredoxins (PRDXs), which also serve to reduce peroxides and are often more abundant in the lens than CAT, to perform daily hydrogen peroxide lens clearance [88,89,90]. These non-enzymatic antioxidants, which include ascorbic acid (vitamin C), tocopherols (vitamin E), and glutathione (GSH), play vital roles; vitamin C is found in the aqueous humor and neutralizes free radicals, vitamin E is present in the cell membrane and prevents the lipid peroxidation of cell membrane, and GSH is a major intracellular antioxidant that regenerates the other antioxidants and directly scavenges ROS [16,91].

### 5.2. Anti-Inflammatory Pathways

Chronic inflammation is associated with various ocular diseases. Oxidative stress and inflammation are linked together as primary to retinal damage. Oxidative stress triggers inflammatory reactions, and inflammation increases the production of reactive oxygen species [92]. Lutein and zeaxanthin are two bioactive compounds that are noteworthy for their ability to reduce the risk of age-related macular degeneration (AMD): in eye tissues, they have antioxidant and photoprotective effects; in the eye, they modulate inflammatory responses. Inflammation activates immune-response facilitators like cytokines, chemokines, and adhesion molecules, exacerbating retinal damage. When redox constancy is interrupted by reactive oxygen species (ROS), retinal cells, including microglia, Müller glia, and astrocytes, produce inflammatory signals. Major proinflammatory markers linked to inflammation-induced retinopathy include several interleukins, factors of the complement system, and chemokines. These mediators contribute to a chronic inflammation that compromises cell barrier defenses, compromising retinal vascular permeability and leading to the degeneration and reduction of cell numbers in retinal layers. Treatment with lutein has shown good results, reversing inflammatory damage and pointedly protecting retinal function in a long-term study in diabetic mouse models by mitigating the effects of elevated vascular endothelial growth factor (VEGF) and restoring proteins such as occludin [93]. Furthermore, lutein and zeaxanthin treatments also counteract photooxidative damage of the retinal pigment epithelium (RPE); changeable damaging protein degradation is a main source of ocular pro-inflammatory mediators and a primary target of photooxidative insult. Oxidative damage of the UPP in RPE may result in ocular inflammation and AMD-related lesions [94]. 

Lutein/zeaxanthin inhibit NLRP3 inflammasome assembly in μglia (50 μM suppresses IL-1β/caspase-1 72% via NF-κB p65 nuclear exclusion), while curcumin (20 μM) blocks IKKβ phosphorylation (Ser181/Thr177) → IκBα stabilization → TNF-α/IL-6 suppression (85%) in high-glucose HRECs. Tissue penetration: Zeaxanthin crosses BRB (vitreous:plasma ratio 0.15), accumulating in the Moller glia to regulate the glienification process in GFAP girosis, whereas polyphenols need to be encapsulated as nanoparticles to achieve therapeutic levels of RPE in the retina (free curcumin < 0.01 μM in vitreous) [95,96].

The eye can deal with inflammation via complex signaling pathways that influence the activation of immune cells and the production of pro-inflammatory factors. The NF- kB and MAPK pathways are central to this process, and the activation of these pathways by the stimulation of the immune system in response to stress or injury results in the release of these cytokines and matrix metalloproteinases (MMPs). Anti-inflammatory measures aim at suppressing such pathways to reduce tissue loss due to inflammation. Also, inflammasomes, or specialized protein complexes, are important for regulating pro-inflammatory cytokines such as IL-1β. Inhibition of the elements of these complexes, including caspase-1, is a viable method of decreasing inflammation and delaying the development of ocular diseases. Ocular health involves a very delicate balance between pro-inflammatory signals and anti-inflammatory cytokines such as IL-10, which assist in down-regulating immune responses and repairing tissues [97,98].

### 5.3. Neuroprotection

**Figure 2:**
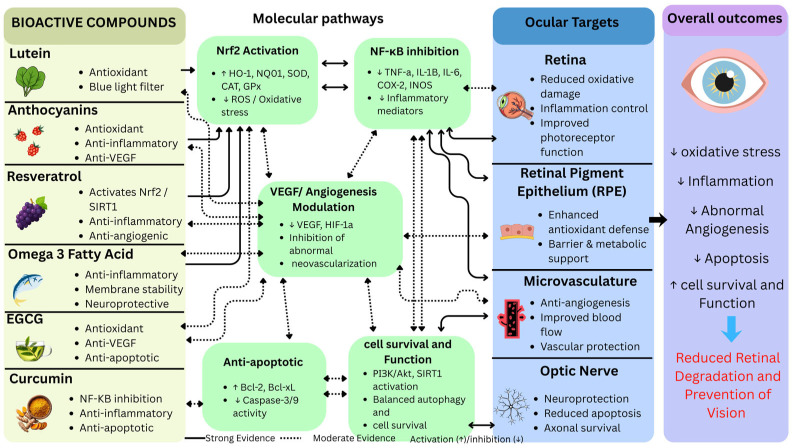
Integrated molecular mechanisms of bioactive compounds in ocular health and retinal degeneration prevention.

Lutein and zeaxanthin allow photoreceptors to localize in outer segment lipid rafts, inactivate singlet oxygen, and inhibit 4-HNE-induced caspase-3 activity, maintaining proteasome and rhodopsin renewal to ensure ONL thickness in light-damage models. Resveratrol, as a polyphenol, stimulates SIRT1 activity by inhibiting p53/FoxO3a and ER stress/CHOP; however, omega-3-derived neuroprotectin-D1 (NPD1) and resolving D1 stabilize mitochondrial membrane potential by Bcl-2 up-regulation and GPR32/ALX signaling, inhibiting cytochrome c release and NLRP3 inflammasome activation in retinal ganglion cells [99,100]. Bioactives have been shown to act as multifunctional neuroprotectants against 60–80% of cell death in AMD, glaucoma and DR degeneration models, supported by the antioxidant defense of POS membranes, neurotrophic BDNF/TrkB support, and antiapoptotic protectants [100]. These neuroprotective mechanisms are further illustrated in Figure 2.

Activation of Nrf2 is causally involved in the RPE shield in AMD (KO increases damage); lutein/zeaxanthin increases macular pigment density, and polyphenols such as resveratrol/quercetin inhibit IL-6/8 through NF-kB. Nrf2-VEGF crosstalk is empirically confirmed in DR, where tricin/curcumin down-regulate the expression of VEGFR2, and carotenoids down-regulate angiogenesis through dh404 to prevent leakage. In the case of glaucoma, associative NF-KB/apoptosis axes are predominant. Lutein/zeaxanthin favors RGC survival via oxidative equilibria, and anthocyanins inhibit neuroinflammatory gliosis. This mapping of disease elucidates the prevalent interventions as opposed to the repetition of compounds [48,96,101,102].

Pinocembrin (25 μM) protects aconitast/α-KGDH TCA cycle enzymes by Erk1/2-Nrf2 in SH- SY5Y (Nrf2 siRNA abrogates), and NPD1 (100 nM) activates RXFP1/GPR32-Bcl-xL up-regulation–cytochrome c retention in RGCs. Pinocembrin (25 μM) induces Nrf2/HO-1 (4.2×) in ARPE-19 via Erk1/2, rescuing aconitase (confirmed by siRNA); curcumin (5 μM) inhibits IKKβ→IL-6↓85% in HRECs. Regarding multimodal lutein distribution in the retina, lutein localizes to the photoreceptor outer segments (lipid raft protection), resveratrol localizes to the ganglion cell layer (SIRT1 nuclear activation), and NPD1 accumulates in the RPE apical membrane, explaining the complementary protection between retinal laminae [87,103,104].

Neuroprotection is the field concerned with protecting against the damage of retinal ganglion cells (RGCs) and other neurons as a result of conditions such as glaucoma, ischemia and metabolic stress. Neurotrophic Factors: Brain-derived neurotrophic factor (BDNF), nerve growth factor (NGF), and ciliary neurotrophic factor (CNTF) are all proteins that are necessary to ensure neuronal survival. They stimulate survival signaling cascades (e.g., TrkA, TrkB, JAK/STAT) that enhance RGC viability and dendritic field preservation. Signaling to survive: In addition to direct growth factor activity, neuroprotection can be achieved by altering intracellular processes, such as calcium homeostasis, autophagy regulation, and glutamate-induced excitotoxicity. Cellular crosstalk: This type of interaction between glial cells (e.g., Muller glia) and neurons is very important; glial cells can release protective factors or clear excessive neurotransmitters to prevent excitotoxic damage of the neural retina [105,106,107].

### 5.4. Pharmacokinetics and Bioavailability

Although in vitro experiments show strong antioxidant activities of polyphenols and carotenoids (e.g., 10–50 μM doses with 50–80% Nrf2 activation or VEGF inhibition), clinical trials indicate poor translation of such activities owing to marked dose response and pharmacokinetic variation. Plasma concentrations are in the low nM range (e.g., lutein Cmax of ~0.1 after 20 mg), not reaching therapeutic concentrations at ocular locations, and have low bioavailability (<5% with curcumin) and clearance, as well as obstacles such as the blood–retinal barrier. Further failures are due to non-selective ROS scavenging (lack of disease-specific targets in mitochondria), patient heterogeneity in baseline oxidative stress, and endpoint heterogeneity (biomarkers vs. clinical vision outcomes), as observed with the small AMD risk reduction of AREDS2. Combinational nano-delivery systems have the potential to help close these gaps by improving ocular delivery by 2–10× [57,108,109,110].

Inter-individual variability has a major role in modulating bioactives in ocular tissues due to genetic polymorphism (e.g., Nrf2 rs6721961/rs35652124 variants change the efficiency of induction, reducing the benefits of lutein by 20–40 percent in inducers), the gut microbiome composition (the Firmicutes/Bacteroides ratio affects polyphenol metabolism to bioavailable urolithins, impacting NF-κB suppression), and metabolic phenotypes (fast vs. slow acetylators influence resveratrol clearance; diabetic hyperglycemia blunts carotenoid RPE uptake). These reasons explain the inconsistent response to AREDS2 (~30% non-response rate) and highlight the need to stratify pharmacogenomics and microbiome-targeted delivery in future RCTs [26,48,111,112,113].

Recent nano formulation technologies provide a dramatic improvement in bioactive compound delivery to the eye by enhancing stability, targeting the retina, and providing controlled release, which overcome the bioavailability problem. Nanocarriers (polymeric/lipid NPs) can result in 5–10× RPE accumulation through RGD-ligands/trans-scleral penetration. Cationic nanocarriers preferentially accumulate in the anterior region, whereas anionic nanocarriers diffuse to the retina to maintain Nrf2 agonists, weeks post-IVT. Hydrophobic carotenoids (e.g., lutein self-emulsifying phospholipid suspensions increase plasma Cmax 11×) are encapsulated as liposomes/niosomes, and topical solubility/permeability enhancers can be enabled in nanoemulsions/micelles (2–3× bioavailability). Translational research (2023–2025) validates the lower frequency and toxicity of injection and the combination of flavonoids/SPMs with AMD/DR; plant-based NPs that are vegan are more in line with sustainable nutraceuticals [114,115,116,117].

Ocular gradients: Lutein reaches 0.1–0.5 μM RPE (20 mg) via LDLR but is negligible in the inner retina; free curcumin < 1 nM vitreous fails BRB. Nano-solutions: RGD-lipid NPs deliver 5.2× RPE curcumin (4-week sustained); self-emulsifying lutein achieves 11× Cmax, addressing tissue thresholds in ARPE-19/Abca4-/- models [34,95].

## 6. Evidence by Eye Condition and Limitations

Certainly, carotenoids, polyphenols, anthocyanins, and flavonoids as natural bioactive compounds show efficacy against age-related macular degeneration (AMD), diabetic retinopathy (DR), glaucoma, cataracts and other ocular disorders. In AMD, carotenoids, which include lutein and zeaxanthin, and anthocyanins have been observed to slow the progression of the disease by increasing the density of the macular pigment and shielding the retinal cells against photooxidative stress. Several clinical trials, such as the AREDS2 study, have shown that lutein and zeaxanthin supplementation can lower the risk of the development of advanced AMD and enhance some visual functioning [118]. The use of polyphenols (quercetin, resveratrol) and some carotenoids can be used to curb hyperglycemia-induced oxidative stress, inflammation, and microvascular damage in diabetic retinopathy (DR). Investigative research demonstrates better endothelial performance and lower retinal vascularity permeability, but there is scant human evidence [99]. In the case of glaucoma and of optic neuropathy, there is limited but promising new evidence. It has been proposed that antioxidant compounds have potential effects that can be used to protect retinal ganglion cells by decreasing the level of oxidative damage and enhancing mitochondrial activity (preclinical models). However, large scale human clinical trials are yet to be conducted. There are some restraining elements surrounding the discussed benefits, such as low and variable bioavailability, the absence of large-scale randomized clinical trials, poor understanding of optimal dosage and duration, and poor performance during the advanced stages of ocular diseases. The interactions between particular bioactive compounds and ocular diseases as well as their mechanism and consequences are outlined in Table 2.

Proposed mechanisms reflect findings from the preclinical and clinical literature. Key outcomes represent reported associations and do not confirm definitive therapeutic efficacy. Evidence strength varies across compounds and disease categories. This table is intended for academic reference and does not constitute clinical guidance. Carotenoids, such as lutein/zeaxanthin, have the best translational preparedness, with RCTs (AREDS2) showing 10–26 percent less advanced AMD development and macular pigment increases, as well as pilot visual functionality improvements, implicating them in clinical AMD prevention. Polyphenols (quercetin/resveratrol) and anthocyanins are at the preclinical/pilot stage, with endothelial protection and neuroprotection of RGCs in DR/glaucoma models but no RCTs as bioavailability limits. New flavonoids (naringenin/pinocembrin) are exclusively pre-clinical (Nrf2/HO-1 mechanistic validation) and requires PK optimization and then human testing. This hierarchy demonstrates the clinical primacy of carotenoids and the dire need to align the strength of the evidence with polyphenol/flavonoid dose-ranging RCTs [46,122,123]. VEGF causality is strong for DR/AMD, as evidenced by clinical blockade and in vitro migration assays. Nrf2 drives cytoprotection experimentally (e.g., KO worsens cataracts/ER stress; rescue prevents). NF-κB/Nrf2-VEGF links are often associative (parallel up-regulation), although Nrf2 KO causally suppresses VEGF-induced migration/tube formation. Although Nrf2 KO causally exacerbates oxidative ocular pathology, NF-κB suppression remains correlative in the absence of ocular specific genetic models. Experimentally validated drivers such as Nrf2 have been reported in cataract versus associative NF-kB roles [48,124,125]. Nanocarriers such as polymeric nanoparticles are associated with better retinal targeting (5–10× RPE accumulation using RGD-ligands) and 3–5× therapeutic index improvements, with sustained trans-scleral/IVT release to provide Nrf2-activating bioactives, reducing toxicity and injection frequency. Liposomes deliver medium-high doses of choroidal/RPE (2–4× vs. free drug) with biocompatibility to combinational flavonoids/SPM delivery; however, vitreous stability is limited. Nano emulsions promote better hydrophobic carotenoid solubility, to achieve topical/scleral delivery (1.5–3× bioavailability), but do not deliver to the posterior segment. In vegan eye health preparations, ligand-functionalized nanocarriers will maximize posterior delivery, whereas emulsions will be used to maximize initial anterior bioavailability, providing a direct response to the poor BRB penetration of polyphenols/carotenoids [58,115,126,127].

## 7. Prospects

Precision nutrition strategies would be the next steps for future study, with simple eye scans, such as macular pigment optical density (MPOD), and genetic markers used to better define personal absorption and metabolic capacity and to personalize supplementation of lutein and zeaxanthin to the individual risk profile for age-related macular degeneration (AMD) and diabetic retinopathy (DR). A genetic-based, metabolically adjusted and microbiome-based precision nutrition model can further maximize the therapeutic efficacy of dietary bioactive molecules in the prevention and treatment of ocular diseases. The delivery of these bioactives via nanocarriers such as liposomes prevents decomposition by stomach, enhancing the delivery of bioactives to the eye tissue by 4–8×, using special receptors of the eye tissue (RPE cells) in targeted therapy. Clinical trials should be performed with large groups to establish the effectiveness of the optimal doses, safety over the long term, beyond the 5 years of data of AREDS2, and efficacy in different ethnicities. Formulas of carotenoids (light protection) + polyphenols (inflammation block) + omega-3s (cell repair signals) have threefold better antioxidant properties. Additional carotenoids, including lutein, zeaxanthin and meso-zeaxanthin, in combination substantially reduce VEGF-induced neovascularization and significantly increase retinal cell survival in animal models [99,118,128,129,130].

To always confirm major Nrf2/IKK2 processes, it is important to adopt a tiered experimental design: Tier 1: ARPE-19 cells are under H_2_O_2_/blue light stress; measure Nrf2 nuclear translocation, IkB stabilization, VEGF inhibition, and the mitochondrial potential (JC-1) of polyphenol + carotenoid combinations (0.1–50 μM). In Tier 2, Abca4-/- retinal explants with light damage are used to assess the level of preservation of the outer nuclear layer, microglial quiescence (Iba1), and macular pigment density following nano emulsion delivery. Tier 3 comprises pilot biomarker studies to monitor plasma urolithins, macular pigment optical density and tear HO-1/NQO1 in Nrf2-genotyped cohorts undergoing standardized vegan formulations (20 mg L/Z + 200 mg polyphenols). This reproducible pipeline can be used to conduct cross-lab meta-analysis and hastens precision RCT design [87,130,131,132].

## 8. Conclusions

The natural bioactive compounds from plants and marine origin show great promise in the field of ophthalmology and for the prevention of the most prevalent eye diseases. Carotenoids (lutein and zeaxanthin), polyphenols (flavonoids and resveratrol), omega-3 fatty acids and some botanical compounds have multiple functions, such as antioxidant, anti-inflammatory, anti-angiogenic and neuroprotective roles. The activities are mainly transmitted via major molecular pathways that are known to play a key role in the pathogenesis of age-related macular degeneration, diabetic retinopathy, glaucoma, and cataracts. Based on available experimental and clinical evidence, it seems that these bioactives can play a role in slowing the advancement of disease by lowering oxidation stress, regulating inflammatory responses, and maintaining the integrity of the cells of the retina. Despite the encouraging results, however, their clinical application is restricted because of their low bioavailability, poor dose standardization, and poor lack of long-term, large-scale randomized controlled trials. Recent advances in delivery systems, like nano-delivery systems and precision nutrition, have emerged as potential solutions to these challenges, with the goal of improving ocular targeting and individualizing therapeutic responses. In the future, clinical trials with well-designed clinical protocols, the optimization of pharmacokinetics, and the incorporation of genetic and microbiome-based stratification should be conducted to fully realize the therapeutic potential of these compounds in the prevention and management of ocular disease.

## Figures and Tables

**Table 1:** Dietary bioactive compounds and their role in the prevention and management of ocular diseases.

Bioactive Compound	Major Natural Sources	Key Mechanisms of Action	Ocular Conditions Targeted	References
Lutein	Spinach, kale, broccoli, corn, egg yolk	Blue-light filtration, antioxidant activity, protection of retinal pigment epithelium	Age-related macular degeneration (AMD), cataracts	[2,26,77]
Zeaxanthin	Corn, orange peppers, goji berries, egg yolk	Enhances macular pigment density, reduces oxidative stress, stabilizes retinal cells	AMD, retinal degeneration	[1,2,77]
Resveratrol	Grapes, berries, peanuts, red wine	Anti-inflammatory effects, activation of SIRT1 pathway, inhibition of oxidative stress	Diabetic retinopathy, glaucoma	[61,62]
Anthocyanins	Blueberries, blackberries, bilberries, purple grapes	Improves retinal blood circulation, antioxidant and anti-angiogenic properties	AMD, diabetic retinopathy	[55]
Epigallocatechin Gallate (EGCG)	Green tea	Reduces oxidative stress, anti-inflammatory activity, protects retinal ganglion cells	Glaucoma, retinal neurodegeneration	[64,65]

Abbreviations: AMD, age-related macular degeneration; EGCG, epigallocatechin gallate; SIRT1, silent information regulator 1. Mechanisms reflect predominant pathways reported across in vitro, animal, and human studies. Evidence levels vary across listed ocular conditions. This table does not constitute clinical dietary advice.

**Table 2:** Role of dietary bioactive compounds in targeted ocular disease management: Proposed mechanisms and key outcomes.

Eye Disease	Key Bioactive Compounds	Proposed Mechanisms	Key Outcomes	References
Age-Related Macular Degeneration (AMD)	Lutein, Zeaxanthin, Anthocyanins	Increase macular pigment density, reduce oxidative stress, inhibit retinal inflammation	Slowed AMD progression and improvement in visual performance	[83,119]
Diabetic Retinopathy (DR)	Resveratrol, Quercetin, Curcumin	Anti-inflammatory effects, reduction of oxidative stress, protection of retinal microvasculature	Decreased retinal vascular damage and inflammatory responses	[8,13]
Glaucoma	Epigallocatechin gallate (EGCG), Resveratrol, Flavonoids	Neuroprotection of retinal ganglion cells, reduction of oxidative stress, mitochondrial protection	Protection of optic nerve cells and reduced neurodegeneration	[48,120]
Cataracts	Lutein, Zeaxanthin, Polyphenols	Prevention of protein oxidation in the lens and reduction of lipid peroxidation	Delayed lens opacification and reduced cataract formation risk	[3,12]
Retinal Degeneration	Omega-3 fatty acids, Anthocyanins, Polyphenols	Anti-inflammatory activity, photoreceptor protection, improved retinal blood circulation	Preservation of retinal structure and visual function	[1,121]

Note: AMD, age-related macular degeneration; DR, diabetic retinopathy; EGCG, epigallocatechin gallate.

## Data Availability

No new data were created or analyzed in this study.
